# *Drosophila* Heat Shock Response Requires the JNK Pathway and Phosphorylation of Mixed Lineage Kinase at a Conserved Serine-Proline Motif

**DOI:** 10.1371/journal.pone.0042369

**Published:** 2012-07-27

**Authors:** Rebecca L. Gonda, Rebecca A. Garlena, Beth Stronach

**Affiliations:** 1 Department of Biological Sciences, University of Pittsburgh, Pittsburgh, Pennsylvania, United States of America; 2 Department of Microbiology and Molecular Genetics, University of Pittsburgh School of Medicine, Pittsburgh, Pennsylvania, United States of America; University of Pecs Medical School, Hungary

## Abstract

Defining context specific requirements for proteins and pathways is a major challenge in the study of signal transduction. For example, the stress-activated protein kinase (SAPK) pathways are comprised of families of closely related transducers that are activated in a variety of tissues and contexts during development and organismal homeostasis. Consequently, redundant and pleiotropic effects have hampered a complete understanding of the individual contributions of transducers in distinct contexts. Here, we report on the function of a context-specific regulatory phosphorylation site, PXSP, in the *Drosophila* mixed lineage kinase protein, Slpr, a mitogen-activated protein kinase kinase kinase (MAP3K) in the Jun Kinase (JNK) pathway. Genetic analysis of the function of non-phosphorylatable (PXAP) and phosphomimetic mutant (PXEP) Slpr transgenes in several distinct contexts revealed minimal effects in JNK-dependent tissue closure processes but differential requirements in heat stress response. In particular, PXAP expression resulted in sensitivity of adults to sustained heat shock, like p38 and JNK pathway mutants. In contrast, PXEP overexpression conferred some resistance. Indeed, phosphorylation of the PXSP motif is enriched under heat shock conditions and requires in part, the p38 kinases for the enrichment. These data suggest that coordination of signaling between p38 and Slpr serves to maintain JNK signaling during heat stress. In sum, we demonstrate a novel role for JNK signaling in the heat shock response in flies and identify a posttranslational modification on Slpr, at a conserved site among MAP3K mixed lineage kinase family members, which bolsters stress resistance with negligible effects on JNK-dependent developmental processes.

## Introduction

Cellular responses to environmental cues require the appropriate spatial and temporal coordination of signaling events. In fact, the sensitivity, amplitude, and duration of signaling activity in response to external stimuli can dictate distinct cellular outputs [1], [2], [3]. A classic example is the regulation of cell proliferation versus differentiation by the duration of Mitogen-Activated Protein Kinase (MAPK) signaling in PC12 cells in response to Epidermal or Nerve Growth Factor, respectively (discussed in [4]). In such kinase-based signal transduction pathways, dynamic modulation of the phosphorylation state or localization of transducers and their substrates are important mechanisms to tune a particular response. This can be achieved in part by additional network inputs in the form of feedback or crosstalk. Here we identify a modulatory phosphorylation site in the *Drosophila* mixed lineage kinase, Slipper (Slpr), which is responsive to environmental stress and required for optimal stress signaling.

Upon exposure to stressful conditions like increased temperature, cells experience an accumulation of misfolded and aggregated proteins [5]. Mechanisms to protect against this, and other cellular damage in the event of recovery from the insult, involve the rapid induction of heat shock protein (HSP) expression and activation of MAPK signaling pathways [6], [7]. Together, the activities directed by HSPs and MAPKs can influence the cellular outcome in response to the stress. For example, cell survival may be favored if the damage is contained and reversible, and apoptotic pathways are blunted. Indeed, several studies have demonstrated the direct inhibition of proapoptotic stress signaling and promotion of prosurvival pathways by HSPs (reviewed in [6], [7]). Sustained stress and irreparable damage, on the other hand, might tip the balance permitting cell death [8]. Thus, the cellular response to stress provides another example of how context can influence a dynamic interplay of signaling activities to determine the fate of the cell, and ultimately the organism.

The stress-activated MAPK pathways, surrounding the Jun NH_2_-terminal Kinase (JNK) and p38 MAPKs, are highly conserved transducers of cellular information in response to a variety of distinct signals. Cumulative evidence shows that these pathways are used during animal development, yet they are also inducible to reestablish homeostasis in an unstable environment [9], [10], [11]. Because of their involvement in large-scale reorganization of gene expression profiles in response to stimuli, aberrant stress signaling pathways are often associated with human diseases, making them attractive targets for therapeutic interventions [12], [13]. The p38 branch of MAPK signaling was first identified in hyperosmotic yeast as the HOG1 pathway [14], but it is clear that p38 signaling is evolutionarily conserved to respond to diverse environmental stresses [11], [15]. The *Drosophila* genome encodes three p38 MAPK genes, *p38a (Mpk2)*, *p38b* and *p38c*, which can be activated by the MAPK kinases Mkk3/*licorne* and Mkk4 [16], [17], [18], [19], [20], [21]. Further upstream, several *Drosophila* MAP3Ks, Tak1, Ask1, and Mekk1, have been implicated in p38 signaling [20], [22], [23]. p38 pathway mutants have few developmental defects [18], however individual members are sensitive to various stresses such as high osmolarity, heat shock, oxidative stress, UV radiation, and immune stimulation [21], [24], [25], [26] and impinge upon other essential cellular pathways like the nutrient-sensing and mitochondrial oxidative pathways, in a context-specific manner [26], [27].

The JNK pathway has also been implicated in diverse cellular responses including apoptosis, wound healing, inflammation, and cell proliferation in many multicellular organisms. In *Drosophila*, the single JNK homolog, Basket (*bsk*), is activated by the MAP2Ks Hemipterous (*hep*) and Mkk4 [28], [29]. There are several MAP3Ks that can act upstream in the JNK pathway. These are Mekk1, Ask1, DLK (*wallenda*), Tak1, Takl2, and MLK (*slpr*) [30]. We have characterized Slpr, the *Drosophila* homolog of mammalian mixed lineage kinases (MLKs), as the JNK kinase kinase required for embryonic dorsal closure [31] and other JNK-dependent morphogenetic events throughout development [32]. Whether and to what extent Slpr plays a role in environmental stress response is largely an open question, however it has been shown to mediate epithelial apoptosis in response to loss of prosurvival signaling [33]. Additional functions redundant with other MAP3Ks might be masked, as is the case during larval epidermal wound repair [34]. On the other hand, mammalian MLKs are clearly activated in response to neurotoxic, metabolic, and inflammatory stresses [35], [36], [37] and signal downstream to p38 in addition to JNK [38], [39], [40]. Recently, *Drosophila* Slpr has been implicated in immune responsive p38 signaling in cultured cells, but this link has not been investigated *in vivo* [25].

Like many kinases, MLK activation is regulated by multiple molecular mechanisms and inputs. These mechanisms include relief of inhibition, dimerization, posttranslational modification, scaffolding, proteolysis, and changes in subcellular localization [40], [41], [42], [43], [44]. In one instance, mammalian MLK3 is phosphorylated by JNK as a means of positive feedback [42]. In particular, JNK phosphorylates the serine within a conserved serine-proline motif, regulating the distribution and activity of MLK3. Specifically, the hypophosphorylated form of the protein is reversibly localized to a detergent-insoluble fraction of the cell where it is inactive, whereas activation of the pathway and subsequent phosphorylation of MLK3 by JNK maintains a signaling-competent pool [42]. Thus, positive feedback from JNK to MLK3 allows amplification of a signaling response, though it is not clear under what physiological conditions this feedback is employed. In this report, using a combination of *in vitro* and *in vivo* assays, we characterize this conserved phosphorylation site in *Drosophila* MLK/Slpr and demonstrate that it is required in the context of stress signaling but not necessarily in development. Namely, phosphorylation of a PXSP regulatory site in Slpr is enriched under specific stress conditions and mutation of this site alters stress responsive behavior of flies. Our results implicate Slpr and JNK signaling along with the p38 pathway in heat shock response and we argue that p38 MAPK-dependent modulation of Slpr phosphorylation might function in crosstalk to maintain Slpr-dependent signaling during stress response.

## Materials and Methods

### Fly stocks

Stocks were maintained at 21°C on cornmeal-molasses agar medium. Crosses were raised at 25°C in 50±10% relative humidity. Stocks obtained from the Bloomington Stock Center are noted here with stock numbers and references. *UAS-bsk^K53R^* BL#9311 [45], *UAS-bsk^DN^* BL#6409 [19], *UAS-bsk^WT^* BL#9310 [46], *UAS-hep^act^* BL#9306 [45], *UAS-hep.CA* BL#6406 [47], *lic^GG01785^* BL#19989, *UAS-Dcr-2.D* BL#24651 [48]. Additional stocks include *UAS-p38b^DN^* [19], *Mpk2^1^* (*p38a^1^* null) [24], *hep^1^* [29], *puc^E69^* (*puc-lacZ*) [49]. *slpr^BS06^* has been described [32]. The following RNAi lines were obtained from the NIG-Fly Stock Center of Japan: *Mpk2^5475R-1^*, *p38b^7393R-1^*, *bsk^5680R-1^*. *w^1118^* was used as a control genotype. For constructs under the control of *UAS* sequences, expression is regulated by the presence of the Gal4 transcription factor [50]. So-called ‘Gal4 driver lines’ thus provide distinct temporal and spatial control of expression depending on the driver line selected, for example: *arm-Gal4 (P{w^+mW.hs^ = GAL4-arm.S}11)* BL#1560 mediates ubiquitous tissue expression, *pnr-Gal4 (P{w^+mW.hs^ = GawB}pnr^MD237^)* BL#3039 directs expression in the embryonic dorsal ectoderm and presumptive dorsal adult notum, *69B-Gal4 (P{w^+mW.hs^  = GawB}69B)* BL#1774 regulates expression in the embryonic ectoderm.

### Slpr transgenes and genetic rescue

*UAS-SlprPXAP* and *UAS-SlprPXEP* were created using site-directed mutagenesis by overlap extension [51]. Overlapping primers incorporated an alanine (GCA) or glutamic acid (GAA) codon in place of the serine 512 codon (TCA). The mutant segment was subsequently swapped with the analogous segment of *UAS-SlprWT* [44] in the UASp vector backbone. Transgenic lines were established after injection of DNA by Genetic Services, Inc. (Sudbury, MA). *UAS-SlprWT*, *UAS-SlprAAA*, and *UAS-SKLC^WT^* have been described [44]. Additional DNA constructs, *SKLC^mut^*, *SKLC^2313^*, and *SK*, were amplified by PCR from extant constructs and cloned into the pcDNA3.1 vector for expression *in vitro*. All constructs were sequenced in entirety to ensure correctness.

For rescue of *slpr* mutants to adulthood, we crossed *y^93j^ w^1118^ slpr^BS06^/FM7; arm-Gal4* females by *w^1118^/Y; UAS-slpr-Tg* males and raised the progeny at 21±1°C for moderate transgene expression. Among the progeny, mutant and *FM7* sibling males were counted to quantify relative eclosion rate as an indication of transgene rescue. To avoid inadvertently including non-*FM7* males that arise from non-disjunction of the maternal X chromosome, we counted only *yellow* ^−^, non-*FM7* males. A minimum of three transgenic lines of each genotype was used for the rescue experiments. Significant differences in *mean survival to adulthood* were determined by unpaired Student's *t*-test with Welch's correction for unequal variances. P values are indicated.

### In vitro transcription, translation, and labeling

*slpr* DNA sequences were cloned into the pcDNA3.1 vector and the T7 promoter was used for transcription initiation. ^35^S-methionine-labeled proteins were produced *in vitro* using the TNT® coupled wheat germ extract transcription/translation system (Promega) according to manufacturer's instructions. Labeled proteins were analyzed by SDS-PAGE and autoradiography.

### Phosphatase treatment

*In vitro* translated protein was incubated with 1× λ-phosphatase at 30°C for 90 minutes in PMP buffer (50 mM HEPES, 100 mM NaCl, 2 mM DTT, 0.01% Brij 35) supplemented with 1 mM MnCl_2_ (New England Biolabs). Proteins were subsequently visualized using SDS-PAGE and autoradiography, and dephosphorylation was detected by shifts in electrophoretic mobility. Aliquots of embryonic protein lysates in NP-40 buffer (150 mM NaCl, 50 mM Tris pH 8.0, 1% NP-40) were added to PMP buffer and treated similarly.

### Western Blots

Overnight collections of embryos expressing the transgenes were dechorionated and homogenized in NP-40 buffer with added protease inhibitors to generate a lysate for Western immunoblot. After separation by 8% SDS-PAGE, proteins were transferred to PVDF membrane and immunoblots were performed using the SNAP i.d.® system (Millipore). Mouse anti-HA (16B12, Covance) and mouse anti-β-tubulin (E7, DSHB) were diluted to 1∶300 and 1∶90, respectively, in 3 ml of 0.2% milk/TBST block. Rabbit anti-SlprSH3 was used at 1∶200 [32]. Sheep anti-mouse HRP secondary antibody (Amersham) was used at 1∶3,000. Adult lysates were made by crushing 5–10 adult flies in 2× Sample Buffer and boiling. Phospho-specific antibodies for immunoblotting were rabbit anti-P-p38, used at 1∶300 (Cell Signaling #9211), rabbit anti-P-JNK at 1∶150 (Cell Signaling #9251), and rabbit anti-P-Slpr-S512 at 1∶300 (#R43, Thermo/Open Biosystems Custom Polyclonal Antibody Production). Donkey anti-rabbit HRP secondary antibody (Jackson Immunoresearch) was used at 1∶3,000. Intensity profile plots were generated in ImageJ with the *Analyze>Gel* function and represent grey values associated with the bands in each lane plotted versus distance migrated on the gel. Fold changes in relative levels of endogenous protein upon heat shock were determined from a minimum of four blots. Student's *t*-test was used for statistical analysis.

### Immunofluorescence and imaging

Immunofluorescence of embryos was performed as described [52]. Mouse anti-fasciclin 3 (7G10, DSHB) was used at 1∶40, mouse anti-HA (Covance 16B12) and rabbit anti-β-galactosidase (Cappel) were diluted to 1∶1000. Secondary antibodies, goat anti-rabbit-TxRed and goat anti-mouse-FITC (Jackson ImmunoResearch Laboratories, Inc.), were used at 1∶200. Images were captured by laser-scanning confocal microscopy on an Olympus FV1000 Fluoview compound inverted microscope and assembled in Adobe Photoshop. Brightfield images of adult flies were captured using a Leica DCF300F camera mounted on a Leica MZ16 stereomicroscope.

### Stress treatments

#### Adult heat shock

For testing heat susceptibility of adult flies, stocks or crosses were raised at 25°C. Newly eclosed adults were collected in fresh vials and aged for 3–5 days. At this point, the experimental details for the mutant stocks versus the transgenic crosses were different and noted here.

For the analysis of mutants, adults (gender combined) were transferred to fresh vials and placed at 37.5±1.0°C in a circulating water bath. Every 30 minutes, flies were scored as *unaffected* (if moving normally on the sides or top of the vial) or as *affected* (if sluggish or immobile on the surface of the food). Data from 3–10 separate experiments were combined, plotted as survival curves using Prism GraphPad software, and analyzed by the log-rank test.

For experiments with transgenic flies, 3–5 day old adults from crosses were separated by gender in empty vials and heat-shocked at 37.5±1.0°C. After an initial 20 minutes, surviving adults were scored every half hour. Data from three separate experiments were combined, plotted, and analyzed in Prism GraphPad. Total numbers of adults tested per genotype is noted in the figure legends.

#### Embryo stress treatments

For heat shock experiments, 14-hour egg collections on grape-juice agar plates were performed at 25°C. Up to 100 embryos were then handpicked to duplicate plates and aged for a total of three hours (3–17 hour-old population). Following this, control plates (−HS) were kept at 25°C while experimental plates (+HS) were transferred to a 37°C incubator for three hours (6–20 hour-old population) and then returned to 25°C. After an additional 24 hours, the number of dead embryos was recorded. Three trials per genotype were performed and the total numbers of embryos varied between 145 and 214. Data was displayed as mean percent lethality and the effect of heat shock on individual genotypes was analyzed using Student's t-test with Welches correction for unequal variance when appropriate.

Embryonic protein lysates for Western immunoblots were prepared in NP-40 buffer from similar collection plates shocked at either 37°C (heat shock) or 4°C (cold shock) for three hours (6–20 hour-old population).

UV irradiation was performed by exposing an overnight collection of dechorionated embryos spread on nytex membrane to 120 mJ/cm^2^ 254-nm light in a Stratalinker® 1800 UV Crosslinker (Stratagene). Embryos were allowed to recover in the absence or presence of light for 1 or 3 hours before protein lysates were prepared. The efficacy of irradiation was determined by removing 10–20 embryos from the irradiated sample to a new plate and scoring for death after an additional 24 hours of incubation at 25°C.

### Longevity Determination

With no more than 30 adults in a single vial, flies were maintained at 25°C and scored daily for survival. Flies were flipped to new vials with yeasted food every 3 or 4 days. Data from multiple vials were combined, plotted as survival curves, and analyzed using the log-rank test in Prism GraphPad. Total numbers are given in the legend.

## Results

### Identification of a phosphorylation site in Slpr

**Figure 1:**
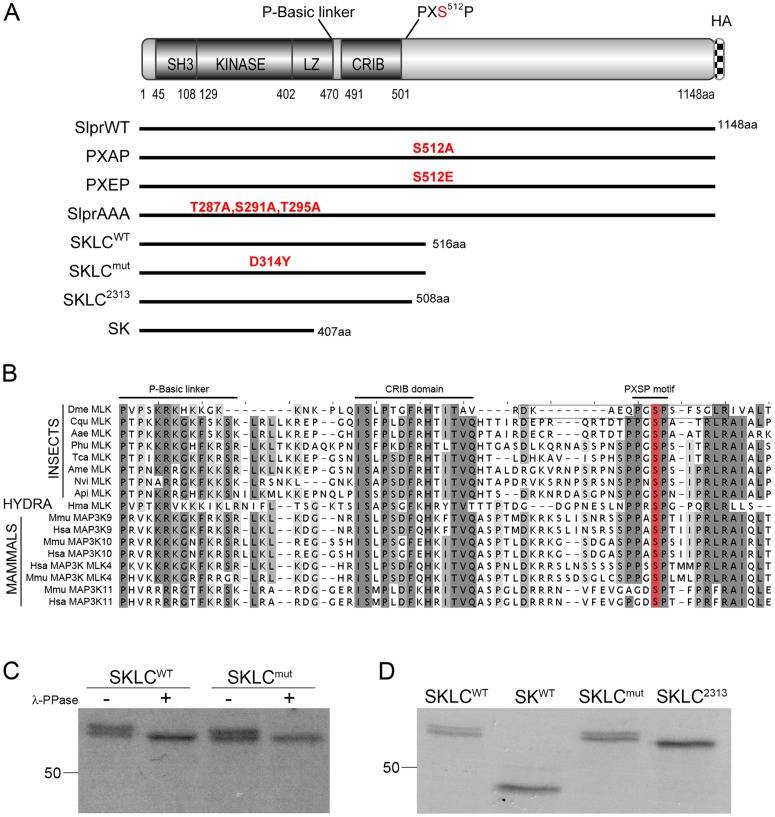
Slpr domain architecture and putative phosphorylation at a conserved PXSP motif. (A) The wildtype Slpr protein (SlprWT) consists of an N-terminal SH3 domain, the catalytic kinase domain, a leucine zipper (LZ) domain, a CRIB domain, and a long C-terminus. Full-length wildtype, mutant, or truncated constructs are indicated by solid lines below. SKLC and SK, encode either the four main domains or the SH3 and kinase only, respectively. SKLC^mut^ contains a kinase mutation, D314Y, while SKLC^2313^ is truncated immediately downstream of the CRIB domain, lacking eight amino acids spanning the PGSP site. SlprAAA contains three mutations (T287A, S291A, and T295A) in the kinase activation loop. PXAP and PXEP are full-length forms of the Slpr protein with an S512A or S512E mutation that either abolishes or mimics phosphorylation, respectively. Each construct has a C-terminal HA tag. The sizes of encoded proteins (excluding the tag) are indicated. (B) Multisequence alignment of insect, hydra, and mammalian MLK family proteins spanning the proline and basic amino acid linker region, the CRIB domain, and downstream conserved PXSP motif. The degree of sequence conservation among the orthologues is indicated by the intensity of highlighting. A phosphorylated form of the peptide in Slpr (Dme MLK) that has been recovered in proteomic studies is boxed. The serine residue targeted for phosphorylation is highlighted in red. (C–D) The different Slpr SKLC proteins and SK were translated and labeled *in vitro* with ^35^S-methionine in a wheat germ lysate. SKLC^WT^ and SKLC^mut^ proteins migrated as doublets by SDS-PAGE. Lambda phosphatase treatment removed the upper band of the doublet of SKLC^WT^ and SKLC^mut^ indicative of a phosphorylated form (C). In contrast, the SK and SKLC^2313^ proteins, which lack the PXSP motif, migrated as single bands (D).

Kinase activation often requires phosphorylation within the catalytic domain, but many kinases have additional regulatory sites. Given that mammalian MLK family members are extensively phosphorylated [53] and that phosphoproteomic studies have identified numerous phosphorylated Slpr peptides [54], [55], we hypothesized that Slpr is regulated by other phosphorylation events outside the kinase domain. In this work, we have characterized a PXSP motif downstream of the CRIB domain (Figure 1) conserved among MLK family members [42] and Figure 1B. We tested the functional consequences of phosphorylation at this site *in vivo* through the use of mutant transgenic constructs.

Using one-dimensional gel electrophoresis to reveal potential modifications on the Slpr protein, we observed that an *in vitro* translated form of Slpr, called SKLC, comprising the N-terminal SH3, Kinase, Leucine Zipper, and CRIB domains (Figure 1A), migrated as two distinct bands by SDS-PAGE (Figure 1C). To determine whether the difference in electrophoretic mobility of the two bands was due to phosphorylation, the *in vitro* translated and labeled sample was treated with lambda phosphatase prior to electrophoresis. Indeed, the doublet collapsed down to a single band upon phosphatase treatment (Figure 1C), indicating that the slower migrating form was phosphorylated. Kinases often undergo multi-step activation in which intramolecular inhibition must be relieved before dimerization or phosphorylation by upstream kinases results in full activation of the protein [41], [56], [57], [58], [59]. Given that SKLC contains the kinase catalytic and leucine zipper domains, we reasoned that homodimerization, via the zipper motifs, might result in kinase autophosphorylation within the activation loop of the kinase domain. To test whether the phospho-form of SKLC was dependent on dimer-mediated autophosphorylation, two additional Slpr constructs were analyzed (Figure 1A): SK, a shorter form of Slpr lacking the leucine zipper and CRIB domains, and SKLC^mut^, which harbors a mutation of an invariant residue in the *α*F helix of the kinase catalytic domain (D314Y). In the context of the full length Slpr protein, the D314Y mutation renders the protein nonfunctional and is presumed to inactivate the kinase [32]. *In vitro* translated SK migrated as a single band (Figure 1D). Notably, SKLC^mut^ behaved similarly to SKLC^WT^, appearing as a doublet before phosphatase treatment (Figures 1C, 1D) and a single band after phosphatase addition (Figure 1C). Together, these results suggest that the phosphorylation of SKLC^WT^ detected *in vitro* is not due to dimerization-induced kinase autophosphorylation, but rather that a kinase in the *in vitro* translation reaction might phosphorylate SKLC.

The phosphorylation of SKLC but not SK spurred us to look for putative kinase recognition motifs in the nonoverlapping region, consisting of the LZ and CRIB domains. Here we noted a PXSP motif, matching the consensus for proline-directed kinases, just downstream of the CRIB domain (Figure 1A,B). Instances of this motif are deeply conserved among MLK homologs (Figure 1B) and it has been shown in HEK293 human embryonic kidney and MCF-7 breast cancer cell lines to positively regulate mammalian MLK3 function upon phosphorylation by JNK [42]. Additionally, two recent phosphoproteomic studies identified this motif in Slpr as an abundant phosphopeptide (Figure 1B, boxed peptide) in cells and embryos [54], [55]. Therefore, we tested whether the PXSP motif accounted for the phosphorylation we had observed in SKLC. To this end, we generated the SKLC^2313^ form, which is shortened by eight amino acids including the PXSP motif (Figure 1A). *In vitro* translated SKLC^2313^ migrated as a single band by SDS-PAGE similar to the lower, hypophosphorylated form of SKLC^WT^ (Figure 1D). The absence of apparent phosphorylation upon deletion of the PXSP motif implies that the serine within this site might be phosphorylated within the context of SKLC^WT^ or that a binding site important for phosphorylation elsewhere in the protein has been eliminated.

### Minor consequences of PXSP phosphorylation during development

**Figure 2:**
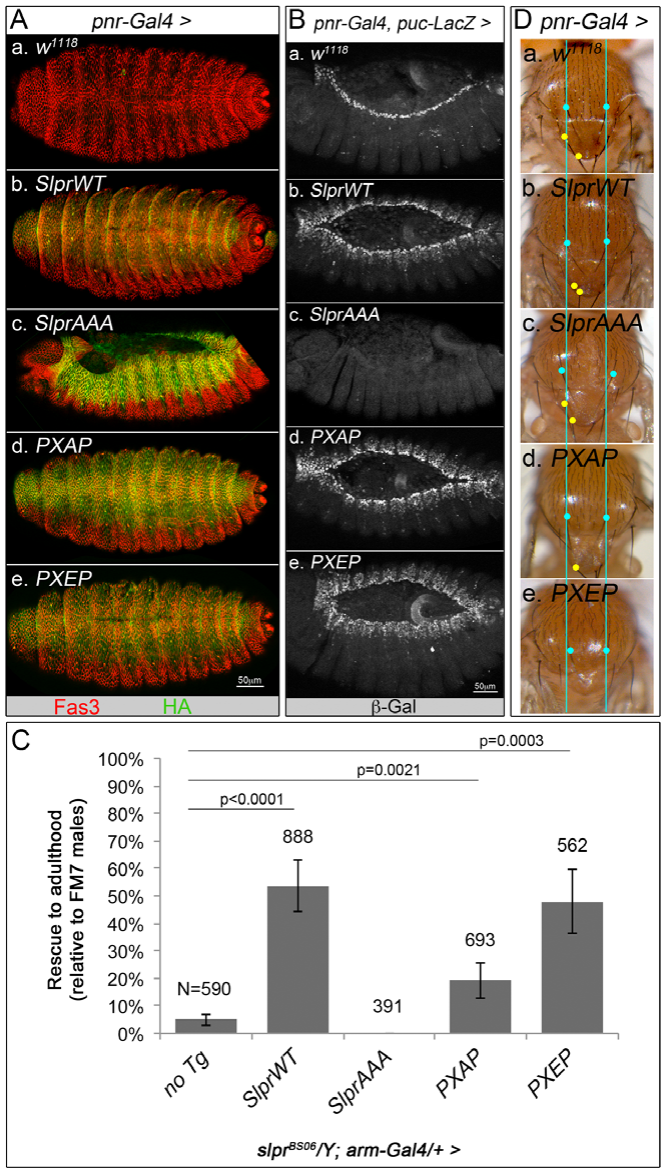
Mutant PXSP transgenes retain signaling function in development. (A) Immunofluorescence staining for Fasciclin 3 (red) in the ectoderm allowed visualization of the progress of dorsal closure in embryos expressing the indicated HA-tagged Slpr transgenes (green) with *pnr-Gal4* compared to a control *w^1118^* (Aa) embryo. Expression of SlprAAA (Ac) causes detachment of the dorsal ectoderm anteriorly and closure defects, whereas overexpression of SlprWT, PXAP, or PXEP, promoted closure of the epidermis at the dorsal midline (Ab,d,e). Images are dorsolateral views of stage 16/17 embryos with anterior left. (B) JNK target gene expression, monitored by the *puc-lacZ* reporter, was upregulated in the *pnr* domain of the embryonic dorsal ectoderm upon expression of all of the indicated Slpr transgenes (Bb,d,e) except the dominant negative SlprAAA (Bc) compared to a control embryo without a Slpr transgene (Ba). Images are dorsolateral views of stage 14/15 embryos, with anterior left. (C) The degree of rescue of *slpr^BS06^/Y* mutants to adulthood, with expression of the indicated Slpr transgenes under *arm-Gal4* control, is displayed as the percentage relative to *FM7/Y* siblings in comparison to a no transgene control. Error bars show s.d. Significant p-values are given, based on Students *t*-test. The total numbers of flies scored are shown above each bar and comprise data from at least three independent transgenic lines per construct. (D) Thorax closure was used to monitor variable levels of JNK signaling during metamorphosis. Blue dots and lines mark the positions of the posterior dorsocentral bristles that flank the dorsal midline. Yellow dots mark scutellar bristles (left of midline only) on the scutellum. SlprWT overexpression with *pnr-Gal4* upregulated JNK activity, which was evident as a narrowed scutellum and/or loss of scutellar bristles (Db) compared to control (Da). Conversely, SlprAAA overexpression impaired JNK-dependent thorax closure resulting in a cleft thorax (note blue dots are farther apart) and widened scutellum. Overexpression of both PXAP and PXEP led to reduced scutellum and loss of scutellar bristles (Dd,e); PXEP resulted in consistently more severe phenotypes. Dorsal views of adult thorax, anterior up.

We have previously implicated *Drosophila* Slpr in JNK-dependent morphological events, such as embryonic dorsal closure, adult thorax closure, male genital disc rotation, and maxillary palp formation [31], [32]. To determine whether modification of the PXSP motif is required for Slpr function during development, we generated nonphosphorylatable (*UAS-PX* ***A*** *P*; S512A) and phosphomimetic (resembling the charged phosphorylated state, *UAS-PX* ***E*** *P*; S512E) transgenes in the context of the full length Slpr protein (Figure 1A). Western immunoblot and immunofluorescence detection of the C-terminal HA epitope tag confirmed expression and localization of the transgenic proteins (Figure S1). As a functional assay for Slpr activity, the progress of dorsal closure was observed upon expression of the transgenes in a wildtype background to determine loss or gain of signaling activity [44]. Using *pnr-Gal4* as an embryonic driver in dorsal ectodermal cells (Figure 2A), we compared the effects on dorsal closure in fixed embryos from overexpression of the following Slpr transgenes: *UAS-SlprWT*, *UAS-PXAP*, *UAS-PXEP* and *UAS-SlprAAA* (a dominant negative form of the protein [44], Figure 1A). Embryos expressing SlprWT completed dorsal closure, similar to control embryos (Figures 2Aa,b), though they were not completely wildtype due to mild upregulation of JNK signaling, which we confirmed using the JNK target gene reporter, *puc-lacZ* (Figure 2B and [32]). In contrast, when SlprAAA was expressed, some embryos failed dorsal closure and displayed defects wherein the embryonic ectoderm (visualized in red with anti-Fas3 immunostaining) detached dorsally (Figure 2Ac), failing to maintain directional progress toward the dorsal midline. SlprAAA expressing embryos also lacked JNK target gene expression at the leading edge (Figure 2Bc). Expression of PXAP did not result in a failure of closure; rather, PXAP-expressing embryos resembled those expressing SlprWT (Figure 2Ad). Like PXAP, expression of the phosphomimetic form, PXEP, promoted dorsal closure (Figure 2Ae), although mild puckering at the midline consistent with upregulation of JNK signaling was noted. Indeed, SlprWT, PXAP, and PXEP overexpression stimulated ectopic JNK pathway signaling, as visualized by reporter gene expression (Figure 2B). Furthermore, overexpression of the same transgenes significantly increased recovery of adult *slpr^BS06^* null mutants, indicative of rescuing function [32], compared to the control group with no transgene expression (Figure 2C). In contrast, the dominant negative *SlprAAA* transgene provided no rescuing function whatsoever, and instead, eliminated recovery of any adult mutant males. Altogether, these data suggest that phosphorylation at the PXSP motif is largely dispensable for Slpr-dependent dorsal closure and viability. We noted, however, that in these and other assays, the PXAP form consistently seemed weaker than PXEP with respect to the magnitude of its effect, and the adult rescue data most clearly exemplify this point. A differential level of expression is not likely to account for the difference in phenotypic potency, given that all of the transgenes were similarly overexpressed and that multiple transgenic lines have been tested with similar results (Figure S1). Importantly, these data demonstrate that the ability to upregulate JNK signaling upon overexpression is not perturbed by the loss of PXSP phosphorylation.

Next, we asked whether phosphorylation at the PXSP motif was required for Slpr function in the context of adult morphogenesis, namely the JNK-dependent process of thorax closure [60], [61]. Using the *pnr-Gal4* driver, it was possible to target transgene expression to the presumptive thorax and recover adults with noticeable dysmorphology of the dorsal notum. The phenotypes followed a similar trend to what we had observed with embryonic dorsal closure. SlprWT, PXAP, and PXEP expression resulted in mild to moderate narrowing of the scutellum and variable loss of scutellar bristles (Figure 2Db, Dd, De), phenotypes that are associated with activation of JNK signaling [44], [62]. Reduced JNK signaling was inferred by the distinctive cleft thorax phenotype and widening of the scutellum seen upon overexpression of the dominant negative SlprAAA protein (Figure 2Dc). These results imply that PXAP and PXEP are functioning to stimulate JNK signaling, like the WT form, in the additional context of adult tissue morphogenesis, similar to what we observed during dorsal closure.

### PXSP phosphorylation in response to stress

**Figure 3:**
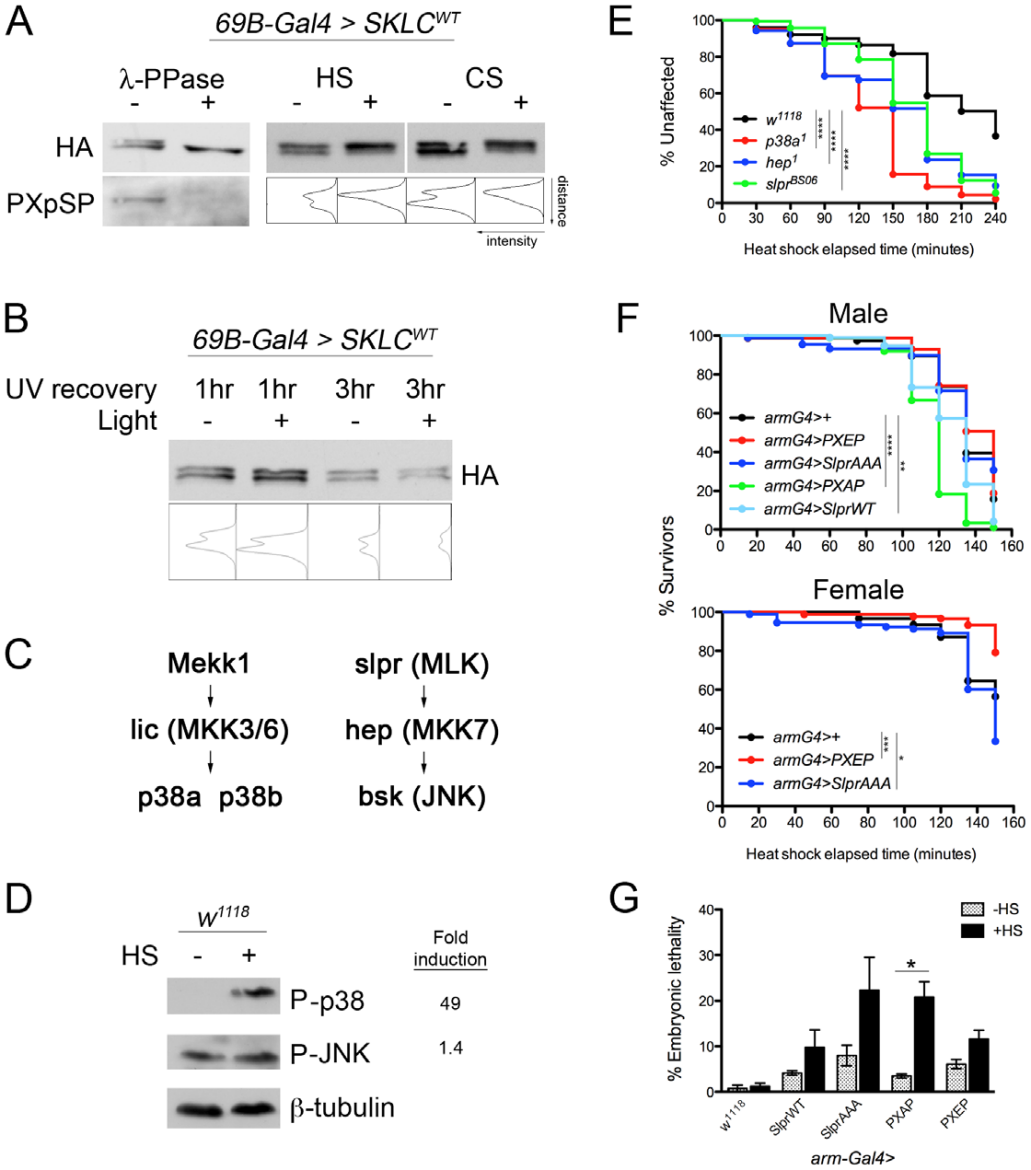
Effects of heat stress on SKLC phosphorylation, stress kinase activation, and survival. (A) HA immunoblot of SKLC^WT^ protein expressed in embryonic epidermis with *69B-Gal4.* Only the upper band of the doublet observed by HA immunoblot was sensitive to lambda phosphatase treatment and was recognized by a S512 phospho-specific antibody (PXpSP). Heat shock (HS) and cold shock (CS) treatments increased the relative levels of the phosphorylated form of SKLC. Intensity profiles are shown with peaks (leftward) corresponding to the upper and lower bands. (B) HA immunoblot of SKLC^WT^ protein expressed in UV-irradiated embryos that had recovered for the indicated time in the absence or presence of light. Pixel intensity plots indicated no enrichment of the phosphorylated form under any condition. (C) Organization of kinases in the *Drosophila* p38 and JNK pathways with mammalian homologs indicated in parentheses. (D) Heat shock induction of phosphorylated stress activated protein kinases, p38 and JNK, in wildtype (*w^1118^*) adults. Fold induction was determined by normalization with β-tubulin. (E) Susceptibility of adult flies mutant for JNK or p38 pathways to continuous heat shock plotted as percent unaffected versus elapsed time in minutes. *w^1118^*, n = 317; *p38a^1^*, n = 267; *hep^1^*, n = 190; *slpr^BS06^*, n = 163. Survival compared to control *w^1118^* animals was significantly impaired for all genotypes by log-rank test. (F) Dry heat shock sensitivity of adult flies expressing Slpr transgenes under the control of a*rm-Gal4*. Upper graph, male cohorts: *armG4>+*, n = 76; *armG4>PXEP*, n = 85; *armG4>SlprAAA*, n = 88; *armG4>PXAP*, n = 87; *armG4>SlprWT*, n = 94. Log rank test: *+* vs. *SlprWT*, p = 0.0023; *+* vs. *PXAP*, p<0.0001. Lower graph, female cohorts: *armG4>+*, n = 62; *armG4>PXEP*, n = 91; *armG4>SlprAAA*, n = 93. Log rank test: *+* vs. *PXEP*, p = 0.0008; *+* vs. *SlprAAA*, p = 0.025. (G) Effect of Slpr transgene expression in embryos subjected to heat stress. Lethality of embryos with the indicated genotypes minus or plus heat shock (HS) was assessed after 24 hours and plotted as the mean percent lethality from three trials. Error bars show s.e.m. Note the increase in lethality of *PXAP* embryos after heat shock compared to untreated siblings, p<0.05 by Student's *t*-test. All other comparisons were not statistically different at a significant threshold.

Since PXSP phosphorylation state does not appear to substantially perturb the developmental functions of Slpr, we hypothesized that this modification might play a role in a non-developmental context, perhaps during cell or organism homeostasis. Indeed, the serine-proline motif resembles the substrate for members of the MAPK family, including the stress-activated protein kinases (SAPKs), JNK and p38. To begin, we first used the mobility of the SKLC form of Slpr by SDS-PAGE as a way to ask whether Slpr modification might be influenced by a stress such as temperature shift. Western immunoblot analysis of lysates from embryos expressing the *UAS-SKLC^WT^* transgene in the developing epidermis with *69B-Gal4* revealed that SKLC migrated as two bands, similar to what we observed after *in vitro* translation of SKLC (Figure 3A, compare to 1C). To verify that the upper band truly represented a phosphorylated form of the SKLC protein, the lysate was treated with lambda phosphatase. As before, the doublet of transgenic SKLC protein was susceptible to dephosphorylation, when in the context of an embryonic lysate (Figure 3A). Furthermore, the phosphorylated form of SKLC was exclusively recognized by a Ser512 phospho-specific antibody (Figure 3A), providing strong evidence of PXSP phosphorylation *in vivo*. This was further verified by examining endogenous Slpr in wildtype embryo lysates (Figure S2). Indeed, we detected a phosphatase-sensitive pool of Slpr using the antibody directed against the phosphor-epitope, PXpSP (Figure S2A). If embryos were subjected to a 37°C heat shock prior to generating the lysates, then the upper phosphorylated form of SKLC became enriched relative to the lower band suggesting increased steady-state levels of the PXpSP form (Figure 3A). Similarly, endogenous phospho-Slpr increased greater than three-fold upon heat treatment, while total Slpr remained constant (Figure S2B,C). Thus, heat treatment induced a shift in the relative levels of the two forms of transgenic SKLC protein and endogenous Slpr. Next, we subjected embryos to a cold shock to address whether the effect was temperature-dependent. Indeed, we noted a similar enrichment of phospho-SKLC upon 4°C cold shock treatment (Figure 3A), suggesting that the phosphorylation state of the PXSP site on Slpr was specifically responsive to temperature stress.

Given that heat shock can induce apoptosis [63], we tested whether the increase in phosphorylated SKLC was simply due to activation of an apoptotic program or an alternative stress condition. So we exposed embryos to UV irradiation, which has been linked to both apoptosis [64] and JNK activation [65]. UV irradiation did not stimulate an increase in the phosphorylated form of SKLC relative to the lower migrating band at either one or three hours of recovery (Figure 3B). Together, these results underscored the specificity in stress response for the modification of Slpr.

The enrichment of phospho-PXSP in response to temperature shift and the resemblance of the peptide sequence to MAPK substrate motifs led us to ask how heat shock affects the stress-activated p38 and JNK MAPK pathways in *Drosophila*, as depicted in Figure 3C. Specifically, we addressed two additional questions experimentally: first, whether *Drosophila* p38 and Bsk/JNK proteins are phosphorylated after heat treatment using phospho-specific antibodies directed against the active form of the kinases and second, whether animals with mutations in these proteins or their activators are compromised in their response to heat shock. Regarding the first question, immunoblots of lysates prepared from adult *Drosophila* revealed robust induction of p38 phosphorylation upon heat shock (Figure 3D), as observed in previous studies [19], [23]. By comparison, induction of Bsk/JNK phosphorylation was minimal, which might reflect the presence of a substantial amount of phospho-JNK protein in the untreated lysate (Figure 3D). Nonetheless, these results demonstrated that under stress conditions, both kinases were present in an active phosphorylated state. Given that high temperature appears to modulate the relative intensities of the two Slpr SKLC forms on an immunoblot, it is at least feasible that JNK or p38 could target Slpr for phosphorylation at the PXSP site under high temperature conditions.

Second, is there any evidence of a requirement for these pathways in the *Drosophila* heat shock response? As mentioned, *p38a* and *p38b* mutants have been shown to be more susceptible to prolonged high temperature than wildtype flies [24], [26], [27], and at least one of the putative upstream activators of the MAP3K class, Mekk1, has been implicated in p38 pathway activation under thermal stress conditions [23], [66]. The role of the JNK pathway in heat shock response in flies is less clear. To this end, adult flies were subjected to a sustained 37°C heat shock to assess their ability to withstand environmental stress, with *Mpk2* (*p38a^1^)* mutant and wildtype flies for comparison [24]. Figure 3E shows the results. Control *w^1118^* flies were gradually affected by the thermal stress such that by 4 hr, more than 60% of the population was sluggish or unresponsive. In comparison, *p38a* null flies became catatonic and unresponsive more rapidly, with nearly 100% of the animals affected after 4 hours. To test whether Slpr was required for this stress response, *slpr^BS06^* null mutants were also subjected to the heat stress and showed sensitivity intermediate between control and *p38a* mutant flies. Given that the JNK pathway has not been implicated previously in heat shock response in flies, we tested another member of the JNK pathway, the JNK kinase, Hep (Figure 3C,E). Like *slpr* mutants, *hep^1^* mutants were more sensitive to heat shock relative to control flies, yet not as severely affected as the *p38a* mutants (Figure 3E). Together, these data demonstrate that JNK and p38 pathway mutants with impaired signaling functions succumb more rapidly to heat stress implying that both pathways are required for heat shock response.

Given that *slpr* mutants show increased sensitivity to heat shock, we predicted that overexpressing Slpr might confer some resistance to thermal stress. In addition, an overexpression assay would allow us to examine the consequences of phosphorylation for heat shock response upon expression of the other mutant transgenes. Using *arm-Gal4* as a ubiquitous driver, we raised flies at 25°C and separated them by gender. After aging the flies for several days with food, we subjected cohorts to heat shock in empty vials and scored survival. The resulting data are shown in Figure 3F. For male flies, providing SlprWT function did not improve their stress response compared to control flies; in fact, survival was impaired. Females did not eclose in sufficient quantity to test. Somewhat surprisingly, flies expressing SlprAAA were either not significantly different (males) or only slightly different (females) in their response to thermostress. Though overexpression of wildtype or dominant negative Slpr had little or no effect on adult response to heat in our assay, we observed greater differences in heat susceptibility among those flies overexpressing the PXAP or PXEP transgenes compared to controls. For example, male flies expressing PXAP were significantly more susceptible to heat shock than controls (Figure 3F, upper graph, green curve), and although the difference between wildtype and PXEP-expressing males was not statistically significant, the median survival of the PXEP cohort (150 minutes, red curve) was one timepoint later than wildtype (135 minutes, black curve). Amongst female flies, a reduced eclosion rate precluded testing PXAP transgenic flies; however, we observed that PXEP expression conferred a significant advantage over control flies in their tolerance of heat treatment (Figure 3F, red vs. black curve). Given that a glutamate substitution does not fully mimic phosphorylated serine, we might expect these results to be even more robust with a serine to aspartate mutant (S>D).

Altogether, these data demonstrate that p38 and JNK signaling are both required for heat shock response, that heat shock enriches for the phosphorylated PXSP site, and that expression of Slpr PXSP mutant transgenes can modulate the sensitivity of adults to heat stress. Thus, a model emerges whereby Slpr signaling becomes important under stress conditions, perhaps to maintain JNK signaling, which is required for optimal stress response. To ensure Slpr signaling during stress, phosphorylation at the PXSP site might be necessary.

### Developmental response of embryos to thermal stress

Given the observations that PXAP and PXEP expression altered the response of adults to environmental heat stress in opposite ways but supported the progression of several developmental processes similar to wildtype Slpr, we reconsidered whether embryos expressing the PXSP mutant transgenes would be able to sustain developmental dorsal closure under stressful conditions. If PXSP phosphorylation is required for Slpr to propagate signaling during stress, we predicted that loss of this phosphorylation might lead to reduced JNK signaling and an increase in embryonic lethality. To this end, we collected embryos ubiquitously overexpressing Slpr transgenes with the *arm-Gal4* driver and exposed half the population to heat shock. In general, heat treatment of embryos resulted in a slightly increased percentage of embryonic lethality compared with untreated siblings (Figure 3G); however, the difference was statistically significant in only one case. Embryos expressing PXAP and subjected to heat stress were nearly six times more likely to die, whereas in the other individual genotypes, *SlprWT*, *SlprAAA*, and *PXEP*, the fraction of dead embryos was increased less than three-fold. Altogether, these data suggest that stress can modulate the levels of PXSP phosphorylation, which in turn provides a mechanism for buffering Slpr function in response to the stress, while it has minimal impact in developmental contexts. The hypophosphorylated state correlated more readily with loss of functional activity, while the hyperphosphorylated form was less sensitive to loss of signaling activity under stress, and showed mild protection.

### Effects of PXSP phosphorylation on longevity

Though many factors determine an organism's lifespan, it is generally accepted that aging represents a gradual loss of optimal molecular, cellular, and ultimately, organismal function. Indeed, activity of the stress-responsive JNK and p38 pathways are correlated directly with longevity in flies and worms [26], [67] suggesting that cellular pathways acting to mitigate stress provide protection over a lifetime. It follows then that impaired stress response could shorten lifespan. Consistent with this idea, several members of the JNK signaling pathway have been implicated directly in lifespan determination in *Drosophila*. For instance, loss of *puc* phosphatase, a negative feedback regulator of Bsk/JNK, results in lifespan extension, which can also be achieved by overexpression of Hep, the upstream activating kinase for Bsk/JNK [68]. The opposite result, shortened lifespan, is observed with *hep* mutants, which have impaired JNK pathway activity [68].

**Figure 4:**
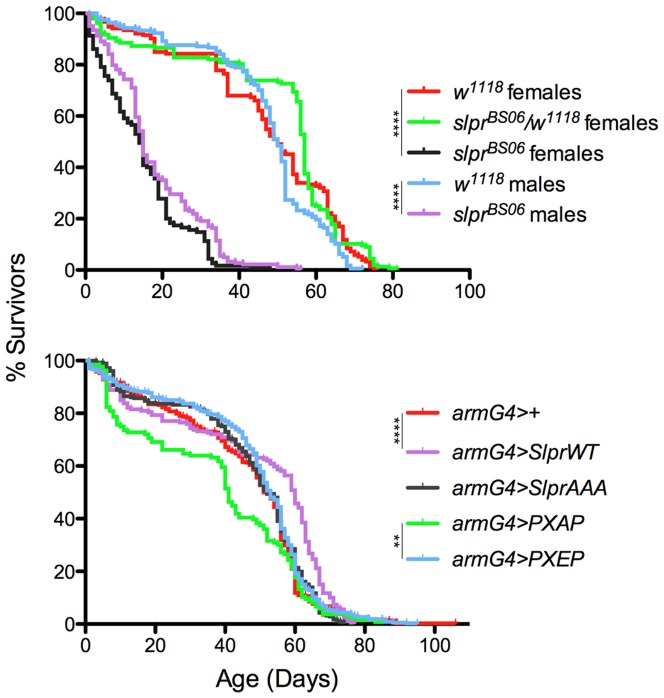
Longevity of *slpr* mutant and transgenic flies. Survival of adult flies was monitored daily. Statistical analysis was performed using the log rank test. Cohort sizes and p-values were as follows. Upper graph: *w^1118^* males (n = 194) vs. *slpr^BS06^* males (n = 183) p<0.0001, *w^1118^* females (n = 153) vs. *slpr^BS06^* females (n = 115) p<0.0001, *w^1118^* females vs. *w^1118^/slpr^BS06^* females (n = 157) p = n.s. Lower graph: Data for males and females is combined. *+* (n = 336) vs. *SlprWT* (n = 184) p<0.0001, *+* vs. *PXAP* (n = 134) or *SlprAAA* (n = 280) or *PXEP* (n = 368) were not significantly different. *PXAP* vs. *PXEP* p = 0.0013.

Using both loss- and gain-of-function mutants and transgenes, we tested directly whether Slpr, one of several upstream activating kinases for Hep in *Drosophila*, would similarly affect lifespan. First, we monitored survival of hemizygous or homozygous *slpr^BS06^* mutants, in comparison to their heterozygous siblings and the progenitor line, *w^1118^*, in which the *slpr^BS06^* mutation was generated. As shown in Figure 4, *slpr* mutants had a significantly reduced lifespan, with a median survival of 14–15 days compared with the control populations, whose median survival was 50–57 days. These data suggest that Slpr activity is a determinant of longevity. To investigate further the potential role of Slpr in lifespan determination, we asked whether the stress-sensitive phosphorylation of Slpr PXSP might play a role in modulating longevity. To address this question, we monitored survival of adults expressing various Slpr transgenes under the control of the *arm-Gal4* driver in an otherwise wildtype background. None of the test populations, including *arm-Gal4>SlprAAA* flies, suffered a reduction in lifespan of the same magnitude as the *slpr^BS06^* mutants (Figure 4). Yet there was a significant increase in median survival age from 52 days for the *arm-Gal4/+* control group to 60 days for the group overexpressing *SlprWT* consistent with previous studies showing that an increase in JNK pathway activity can extend lifespan [68]. Notably, although the survival curves for either PXAP- or PXEP-expressing flies did not differ significantly in comparison to the control population expressing no transgene, survival of the PXAP population versus the PXEP population was significantly different from each other, with median survival age of 41 days and 53 days respectively (Figure 4). Thus, the inability of the PXAP transgenic protein to be phosphorylated was a deficit to animals expressing it, with respect to lifespan and stress response.

### Requirements for PXSP phosphorylation in vivo

**Figure 5:**
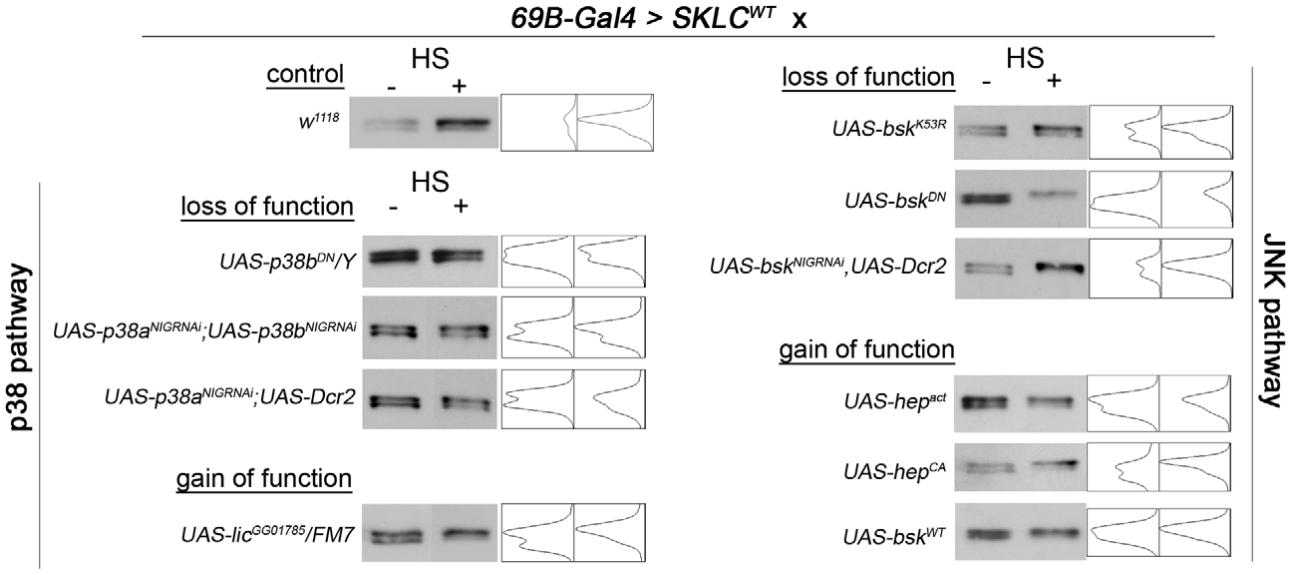
Assessing MAPK contributions to SKLC phosphorylation. HA immunoblots of lysates from embryos programmed to express SKLC^WT^ with the *69B-Gal4* driver (control), or with either loss-of-function (RNAi knockdown or dominant negative) or gain-of-function (wildtype or constitutive active) constructs for the p38 or JNK pathways. Each genotype was assayed without and with heat shock (HS) treatment to test the requirement for heat-induced phosphorylation. The intensity (grey value) profiles are shown with peaks (leftward) representing the upper and lower bands of SKLC protein.

Because phosphorylation within the PXSP motif of Slpr provided some protection against heat stress, we hypothesized that the responsible kinase might be active in stress conditions to propagate the appropriate response. We turned our attention to the SAPKs, JNK and p38, which are proline-directed kinases that could phosphorylate the serine within the PXSP motif of Slpr as a means of feedback or crosstalk. To determine if one of the SAPKs phosphorylates PXSP in the fly, *UAS-SKLC^WT^* was expressed in the embryonic ectoderm using *69B-Gal4*, and the relative level of the phospho-form was assessed after coexpression with constructs that modulate the SAPK pathways (Figure 5). For instance, if JNK is responsible for SKLC phosphorylation, then it is expected that a corresponding change in the mobility of SKLC by SDS-PAGE will be observed with activation or inhibition of Bsk or its upstream activator, Hep. In the absence of heat shock, in every case where SKLC was coexpressed with loss- or gain-of-function constructs for the p38 or JNK pathways, there were two conspicuous forms of SKLC. No perturbation resulted in loss of the phospho-form of SKLC, nor was there a substantial increase in the relative levels of the phosphorylated form upon coexpression of gain-of-function constructs for the SAPK pathways, Lic (p38), Hep and Bsk (JNK).

Given that heat treatment shifts the relative amounts of SKLC toward the phosphorylated form, we reasoned that exposure of embryos to heat shock might increase the activity or requirement of the putative PXSP kinase and sensitize the system to knockdown or interfering activities from coexpression. Indeed, the relative intensity of the phosphorylated form of SKLC was increased after exposure to heat, such that the intensity profile appeared as one peak (Figure 5, control). This enrichment was prominent with all of the loss-of-function experimental constructs; however, the magnitude of the enrichment was substantially reduced upon simultaneous block or knockdown of the p38 kinases. This was seen as two residual peaks in the intensity profiles in the presence of heat shock. The results obtained from these *in vivo* studies most strongly implicate a requirement for p38 MAPK function in PXSP phosphorylation in response to heat stress of the embryo.

## Discussion

In this report, we describe a previously unidentified regulatory mechanism for MLK/Slpr activity in *Drosophila*. While the role of Slpr in JNK signaling during dorsal closure has been well studied, there has been little evidence to date that Slpr is required for stress response *in vivo*. This is the first demonstration that Slpr is modified in response to stress. Specifically, phosphorylation within a conserved PXSP motif of Slpr is enriched upon heat or cold treatment and modulates organismal response to thermostress.

Based on the appearance of two forms of SKLC in lysates prepared from *in vitro* and *in vivo* sources, and the detection of endogenous Slpr in embryos with a phospho-specific antibody, we conclude that under steady state conditions, basal phosphorylation and dephosphorylation of the PXSP site must be occurring. Upon heat shock, p38 kinases were strongly activated (Figure 3 and [19], [69]) and the prevalence of the phosphorylated form of PXSP was increased in the endogenous protein over three-fold. In addition, the heat shock-induced enrichment of phospho-SKLC was diminished in embryos where p38 activity was compromised in the epidermis by RNAi or dominant negative constructs. Thus, while the coincidence of these observations is consistent with a direct relationship, wherein p38 MAPK phosphorylates serine 512 in the PXSP motif upon stress treatment, *in vitro* IP-kinase assays with mammalian p38α (Stronach, unpublished data) do not support that interpretation, and alternative explanations are conceivable. For example, other kinases are activated by p38 MAPKs, including the MAPKAP/MK, MSK, and MNK family members [15], which might be responsible for PXSP phosphorylation. Moreover, the enrichment of phospho-PXSP upon temperature shift might not necessarily be due to increased phosphorylation by an active kinase, but rather, loss of phosphatase activity. Thus, it is interesting to note that in several mammalian tissue culture systems, dual-specificity MAPK phosphatases are heat labile, accounting in part for the accumulation of phosphorylated MAPKs to sustain signaling under stress [70], [71], [72]. Whether this mechanism is conserved in *Drosophila* has not yet been investigated; however, a recent study revealed that the MAPK phosphatase, Puckered, was phosphorylated by JNK and p38 in response to oxidative stress, though the consequences of this modification are still poorly understood [73]. Nevertheless, inactivation of MAPK phosphatases could contribute indirectly to the observed increase in phospho-Slpr. Mechanistically, reduced dephosphorylation of a MAPK that normally provides for the basal level of phosphorylation on PXSP, might secondarily enhance this modification, as observed *in vivo*. Also, the observation that phospho-SKLC was not enriched in lysates from UV-treated embryos, in which p38 is expected to be active, argues against a general p38 requirement and perhaps in favor of a temperature-dependent mechanism.

Though the details of how p38 MAPKs are involved in regulating Slpr phosphorylation under stress conditions remain to be elucidated, the requirement for p38 MAPK signaling in *Drosophila* heat shock response is clear (Figure 3 and [24], [26], [27]). Our results also define a requirement for JNK signaling in response to heat stress. Indeed, both *slpr* and *hep* mutants are more sensitive to prolonged heat shock than control animals. While this result is not that surprising in light of transcriptional profiling studies demonstrating that targets of both JNK and p38 pathways include genes essential for stress response, such as HSPs [25], [68], [74], it does contrast with experiments demonstrating that HSP-mediated inhibition of JNK signaling is protective to cells providing a mechanism for acquired thermotolerance [7], [70]. The nature of the experimental systems, cells versus whole organism, or the duration of stress, minutes versus hours, may account for this potential discrepancy in the role of JNK activity in heat stress response. Our findings here, show that loss of either JNK or p38 signaling pathways under heat stress conditions impairs the necessary response, making animals susceptible to prolonged injurious insult. Although phosphorylated active Bsk/JNK was detected in our lysates even under non-stress conditions, possibly accounting for the basal levels of PXSP phosphorylation, we observed no impact on phospho-SKLC when JNK pathway activity was altered positively or negatively. Nor did we detect phosphorylation of SKLC by JNK1 *in vitro* (not shown). Taken together, it seems unlikely that feedback regulation of Slpr by Bsk/JNK could account for the temperature dependent enrichment of PXpSP even though the precedent of mammalian MLK3 phosphorylation by JNK as a positive feedback mechanism has been described [42].

What then is the physiological purpose of the conserved PXSP site? And does the enrichment of the phosphorylated form upon high or low temperature shock have any consequences *in vivo*? In developmental processes that require Slpr-dependent JNK signaling, we found that mutants in the PXSP site behaved more or less like wildtype Slpr. When overexpressed, the PXAP and PXEP proteins were competent to upregulate JNK signaling in the embryo and to complement a *slpr* mutant to viability to variable extents. However, when expressed in animals that were also subjected to heat stress conditions, there was a deficit in the response of animals expressing the non-phosphorylatable form, PXAP, and a measurable protection in animals expressing the phospho-mimetic PXEP protein. Moreover, flies expressing the PXAP form had a shorter median survival value at 41 days relative to controls with 51-day median survival in longevity experiments. So flies having this modification (PXEP), or being able to dynamically regulate it (PXSP), were at an advantage relative to flies expressing a form that could not be modified (PXAP), suggesting that phosphorylation at this site is functional and not just a byproduct of heat shock.

The lower overall recovery of flies expressing PXAP transgenes might indicate that they are generally less fit than other genotypes used here. While this is a concern, the results we obtained using several independent transgenic lines provides evidence against a spurious effect on fitness due to the insertion site interfering with an unknown gene. Moreover, the results that PXAP expression in the embryo had a minimal impact on embryonic lethality or signaling under nonstress conditions, coupled with the evidence that UV radiation does not appear to elicit changes in the phosphorylation status at PXSP, as does heat shock, suggests a distinct, physiological response.

While our data suggest a role for PXSP phosphorylation in thermostress signaling, it remains unclear by what mechanism this modification modulates Slpr activity. To probe the effect on Slpr when PXSP phosphorylation is lost, we performed several biochemical experiments (not shown). Proteolytic analysis of PXAP and SlprWT revealed similar proteolysis patterns with or without heat shock, indicating analogous protein folding between the two forms, thus ruling out the notion that the PXAP protein is grossly misfolded. Consistent with that observation, the unfolded protein response pathway was not induced in embryos overexpressing PXAP under non-stress conditions. Furthermore, we were unable to detect a biochemical interaction between Slpr and HSP70 or HSP90 under normal or stress conditions. Thus, while phosphorylation within the PXSP motif might not be required for proper protein folding, other explanations are credible, including regulation of protein turnover, spatial distribution, or activity. JNK phosphorylation of mammalian MLK3 at the serine-proline site regulates its distribution in the cell to modulate signaling intensity [42]. We are currently examining the localization of Slpr transgenic proteins under different conditions to explore this possibility. Alternatively, phosphorylation within this motif may affect binding of substrate or upstream effectors. Tests are ongoing to determine the consequences of PXSP phosphorylation at the protein level.

Another question raised by our results is which tissue requires active JNK or p38 signaling during adult heat stress? Accumulating evidence suggests that the nervous system, in particular insulin producing neurosecretory cells, upregulate JNK signaling during oxidative stress response, which counteracts insulin/IGF signaling allowing an adaptive systemic response [75], [76], [77], [78]. Additionally, the JNK pathway is normally active in neuronal development, particularly in the mushroom body [2], and in maintaining neuronal homeostasis in both *Drosophila* and mammals [79], [80], [81]. We have observed that *arm-Gal4* directs transgenic Slpr protein expression weakly in larval imaginal discs and nonneural tissue, but strongly in the larval and adult nervous system, primarily in the mushroom bodies of the brain [44]. Intriguingly, this structure has recently been linked to a systemic response of adult flies to heat shock, in animals deficient for the mitochondrial phosphatase PGAM5 [82]. Moreover, this phosphatase has been shown previously to regulate the activity of ASK1, a MAP3K in the JNK signaling pathway [83]. Whether *arm-Gal4* directed expression of Slpr transgenes in the insulin-producing cells of the adult brain or in other structures including the mushroom body is responsible for the phenotypic differences that we observe in adult stress response will be a topic for future investigations.

The results presented in this report demonstrate that the JNK pathway, including Slpr, is required for heat shock response. Modification of the PXSP site in Slpr is enriched in a temperature dependent fashion and correlates with the degree of susceptibility to heat stress. We argue that phosphorylation of the PXSP motif is important to sustain JNK signaling in attempt to reestablish homeostasis. The presence of phosphorylated Slpr protein at steady state under nonstress conditions might buffer signaling activity from complete failure in rapidly changing conditions.

## Supporting Information

Figure S1 **Expression and localization of transgenes in embryonic epidermis.** (A) Western immunoblot for the HA tag shows expression of the transgenic proteins in embryonic lysates. Specific transgenic lines are indicated in parentheses. (B) HA-directed immunofluorescence detecting the tagged transgenic proteins expressed under the control of *pnr-Gal4*. Dapi was used as a counterstain. Images are lateral views of stage 14 embryos.
(TIF)

Figure S2 **Enrichment of endogenous Slpr, phosphorylated at the PXSP motif, upon heat shock.** (A) Western immunoblots of embryonic lysates without or with λ-phosphatase treatment. Blot was first probed with anti-Slpr PXpSP antibody (P-Slpr), then stripped and reprobed with anti-Slpr SH3 antibody (total Slpr). (B) Western blot with the indicated antibodies probing amounts of protein before and after heatshock (−,+HS). (C) Quantification of phospho-Slpr versus total Slpr levels upon heat shock, normalized to β-tubulin as a loading control. The upregulation of the phosphorylated form of Slpr upon heat shock is significantly different than total Slpr (p = 5.6E-06).
(TIF)
