# Supplementary figures and images for: Drosophila Heat Shock Response Requires the JNK Pathway and Phosphorylation of Mixed Lineage Kinase at a Conserved Serine-Proline Motif
Source: PLoS One. 2012 Jul 27;7(7):e42369. doi: 10.1371/journal.pone.0042369 (PMC3407086; doi:10.1371/journal.pone.0042369)

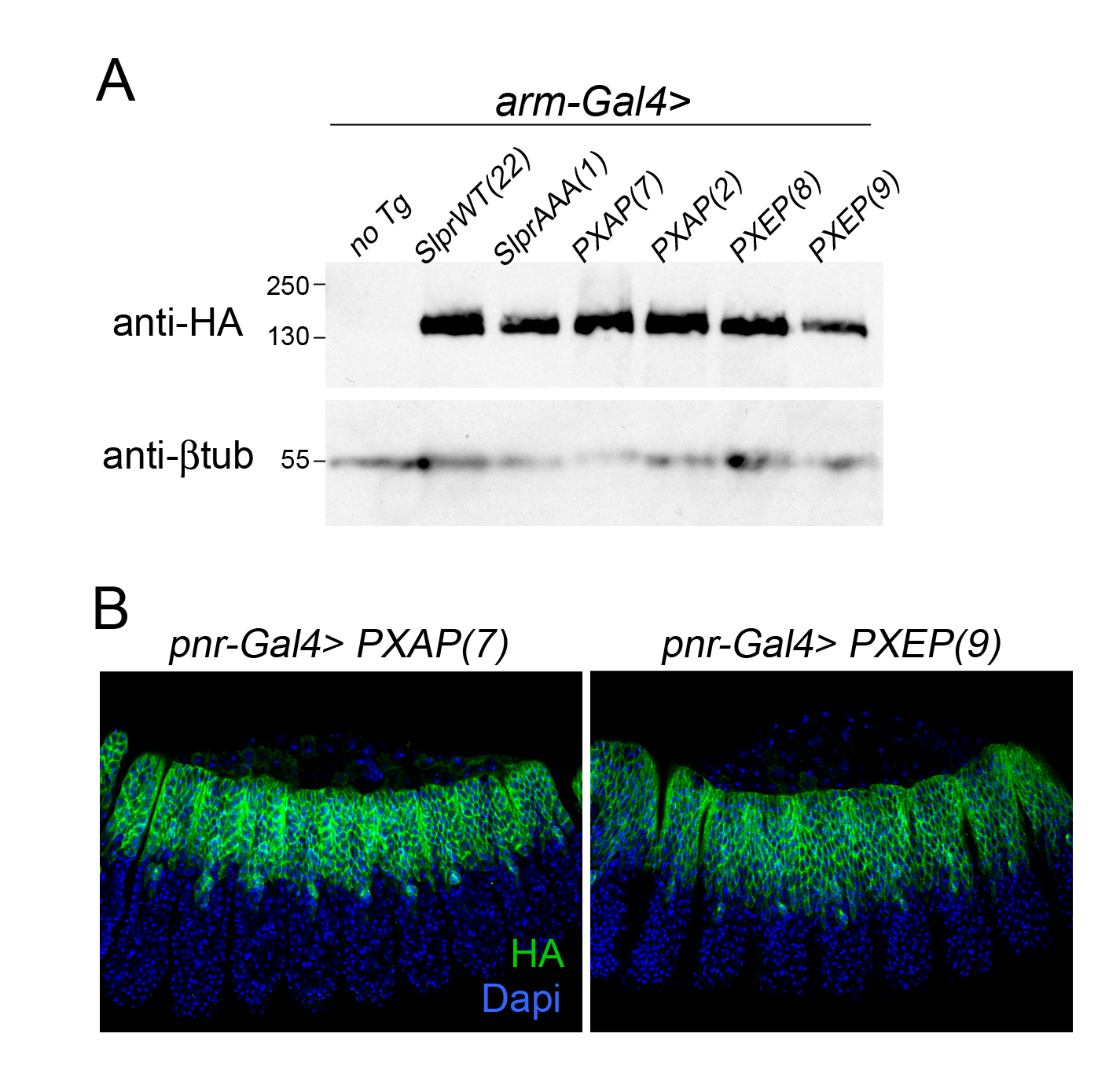

Supplement: Figure S1 — Expression and localization of transgenes in embryonic epidermis. (A) Western immunoblot for the HA tag shows expression of the transgenic proteins in embryonic lysates. Specific transgenic lines are indicated in parentheses. (B) HA-directed immunofluorescence detecting the tagged transgenic proteins expressed under the control of pnr-Gal4. Dapi was used as a counterstain. Images are lateral views of stage 14 embryos. (TIF) [file pone.0042369.s001.tif]

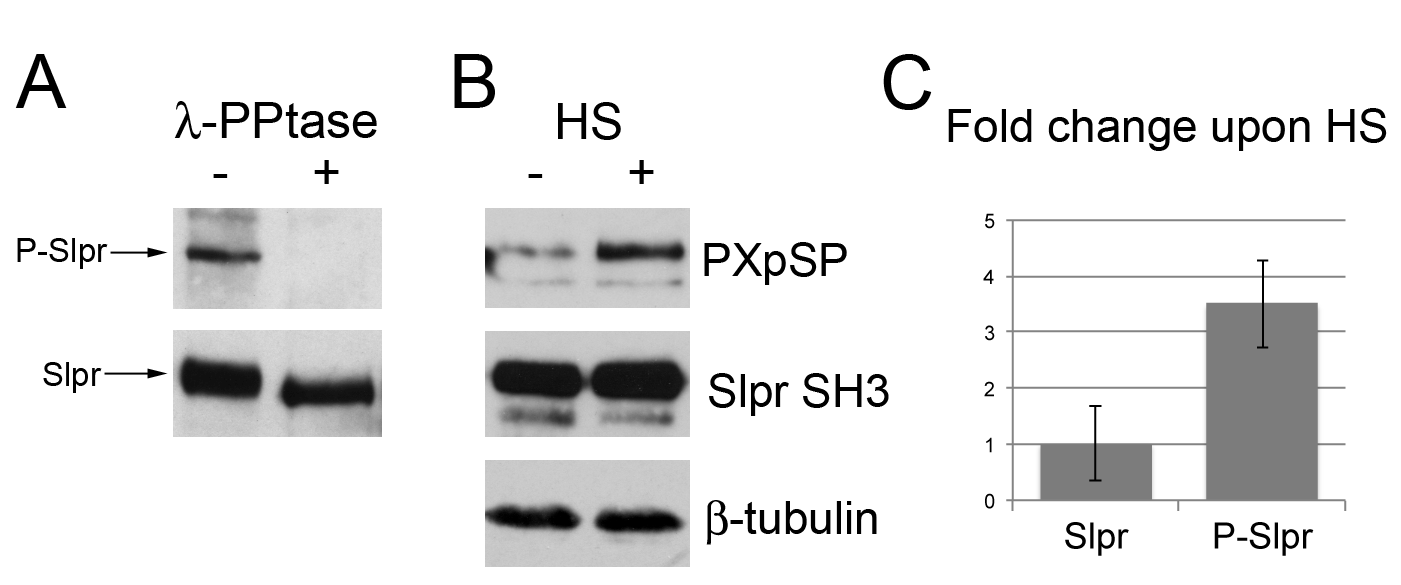

Supplement: Figure S2 — Enrichment of endogenous Slpr, phosphorylated at the PXSP motif, upon heat shock. (A) Western immunoblots of embryonic lysates without or with λ-phosphatase treatment. Blot was first probed with anti-Slpr PXpSP antibody (P-Slpr), then stripped and reprobed with anti-Slpr SH3 antibody (total Slpr). (B) Western blot with the indicated antibodies probing amounts of protein before and after heatshock (−,+HS). (C) Quantification of phospho-Slpr versus total Slpr levels upon heat shock, normalized to β-tubulin as a loading control. The upregulation of the phosphorylated form of Slpr upon heat shock is significantly different than total Slpr (p = 5.6E-06). (TIF) [file pone.0042369.s002.tif]
